# Decoding and Discrimination of Chemical Cues and Signals: Avoidance of Predation and Competition during Parental Care Behavior in Sympatric Poison Frogs

**DOI:** 10.1371/journal.pone.0129929

**Published:** 2015-07-01

**Authors:** Lisa M. Schulte, Martin Krauss, Stefan Lötters, Tobias Schulze, Werner Brack

**Affiliations:** 1 Department of Biogeography, Trier University, Trier, Germany; 2 Department of Effect-Directed Analysis, Helmholtz Centre for Environmental Research, Leipzig, Germany; University of Arkansas, UNITED STATES

## Abstract

The evolution of chemical communication and the discrimination between evolved functions (signals) and unintentional releases (cues) are among the most challenging issues in chemical ecology. The accurate classification of inter- or intraspecific chemical communication is often puzzling. Here we report on two different communication systems triggering the same parental care behavior in the poison frog *Ranitomeya variabilis*. This species deposits its tadpoles and egg clutches in phytotelmata and chemically recognizes and avoids sites with both predatory conspecific and non-predatory heterospecific tadpoles (of the species *Hyloxalus azureiventris*). Combining chemical analyses with in-situ bioassays, we identified the molecular formulas of the chemical compounds triggering this behavior. We found that both species produce distinct chemical compound combinations, suggesting two separate communication systems. Bringing these results into an ecological context, we classify the conspecific *R*. *variabilis* compounds as chemical cues, advantageous only to the receivers (the adult frogs), not the emitters (the tadpoles). The heterospecific compounds, however, are suggested to be chemical signals (or cues evolving into signals), being advantageous to the emitters (the heterospecific tadpoles) and likely also to the receivers (the adult frogs). Due to these assumed receiver benefits, the heterospecific compounds are possibly synomones which are advantageous to both emitter and receiver ‒ a very rare communication system between animal species, especially vertebrates.

## Introduction

Communication in the biological sense is defined by Wilson [1] as the action of one organism that alters the probability pattern of behavior in another. A major difficulty when defining communicative systems is to distinguish between evolved functions and incidental effects [2,3]. Chemical communication is generally regarded as the oldest and most widespread form of communication that occurs at all levels of biological organization [4–6]. Besides chemical signals, which are intentionally released by the sender, receivers also react towards unintentionally released chemicals, which are defined as chemical cues [7]. While chemical signaling is usually advantageous to the sender, releasing chemical cues is either neutral or damaging to it. However, if the reaction of the receiver towards chemical cues is beneficial to the sender, evolution into signaling is expected [8]. The evolution of chemical communication is therefore described as ‘chemical ritualization’ towards chemical signals [7]. Intraspecific signals are defined as pheromones [5], while signals between different species can be divided into synomones and allomones. While allomones are only beneficial to the sender (e.g. being used in predacious or parasitic relationships), synomones are beneficial to both, sender and receiver. That is, synomones are used in mutualistic relationships and are very rarely found between animal species (especially vertebrates) [9]. The proper categorical classification of a chemical substance that triggers a certain behavior in another individual is often puzzling and sometimes only a closer look at the chemical compounds involved brings clarification (for examples see [10–12]).

In this study we combine behavioral in-situ bioassays and comparative chemical analyses in order to categorically classify intra- and interspecific communication systems in two species of poison frogs. Frogs that display advanced levels of parental care behavior (including the choice of exclusive breeding habitats used by various species) offer unique opportunities to investigate the influence of chemical communication on parental decisions. The Neotropical poison frog *Ranitomeya variabilis* [13] uses phytotelmata (such as small water bodies in plant axils [14]) for both egg clutch and tadpole deposition. It attaches its egg clutches just above the water surface and when the larvae hatch the male parent returns and transports them individually into different phytotelmata [15]. Because its tadpoles are cannibalistic, feeding on both conspecific tadpoles and eggs [16,17], parent *R*. *variabilis* need to locate unoccupied phytotelmata for their offspring. The recognition and avoidance of occupied water bodies is triggered by chemicals released by conspecific tadpoles [18]. An identical or even stronger avoidance reaction was shown towards chemicals released by tadpoles of another poison frog species of the same family, *Hyloxalus azureiventris* [19]. The tadpoles of this aposematic species occupy large ground phytotelmata, which sporadically are also used by *R*. *variabilis* for depositing tadpoles. We aimed to identify which chemical compounds trigger the avoidance of both con- and heterospecific tadpoles and to categorize the communication systems behind this behavior.

The avoidance of predatory conspecific tadpoles (i.e. the sender) increases the frogs’ (i.e. the receivers’) reproductive success, but is not advantageous to the sending tadpoles occupying the phytotelmata as they lose a possible food resource. In this case we might define the chemicals that trigger the avoidance behavior as chemical cues, released unintentionally by the tadpoles. However, this might not be the case for the chemicals released by *H*. *azureiventris*. These heterospecific tadpoles are not predatory and should therefore not pose a threat to (but may be in competition with) the offspring of *R*. *variabilis*. But the avoidance behavior of *R*. *variabilis* allows *H*. *azureiventris* tadpoles to evade threats of both predation and competition; so such behavior is presumably advantageous for them. Thus, the chemicals released by *H*. *azureiventris* tadpoles might be signals to the heterospecific adults of *R*. *variabilis* (or cues that are in the process of evolving into signals, see [7,8]). However, the phylogenetic relationship between these two poison frog species (even if not very closely related) raises the question of whether the chemicals released by both species might be the same, meaning that the avoidance of *H*. *azureiventris* might be a result of *R*. *variabilis* not being able to distinguish between con- and heterospecific tadpoles.

Using effect-directed analysis [20] combining in-situ bioassays, fractionation and comparative chemical analyses, we tested the following hypothesis in order to identify and categorize this interspecific communication system:

(i) The chemical tadpole compounds avoided by *R*. *variabilis* are identical between *R*. *variabilis* and *H*. *azureiventris*. This would complicate the classification of the interspecific compounds produced by *H*. *azureiventris* tadpoles: (a) they could be a non-species-specific by-product and might therefore be defined as a chemical cue, not adapted for use in communication. Or (b) they might be produced in order to mimic the compounds of *R*. *variabilis*. In this way the *H*. *azureiventris* tadpoles would use chemical signals to mislead the adult *R*. *variabilis* in order to prevent sharing a pool with their predatory tadpoles. These signals could be defined as allomones, which benefit the *H*. *azureiventris* tadpoles (i.e. the sender) because they are not recognized as interspecific signals, but confused with intraspecific cues by the *R*. *variabilis* adults (i.e. the receiver).

Alternatively, we hypothesize that (ii) the biologically active compounds chemically differ between the two species. This would mean that the compounds released by *H*. *azureiventris* tadpoles can be defined as signals (or cues evolving into signals) that trigger a specific response in the heterospecific receiver (the *R*. *variabilis* adults). The chemicals involved here would be most likely synomones, since they benefit the sender (*H*. *azureiventris*) and are recognized but nevertheless avoided as heterospecific compounds by the receiver (*R*. *variabilis*). A possible benefit for the receiver might then be the avoidance of a competition situation for its own offspring.

## Materials and Methods

**Fig 1:**
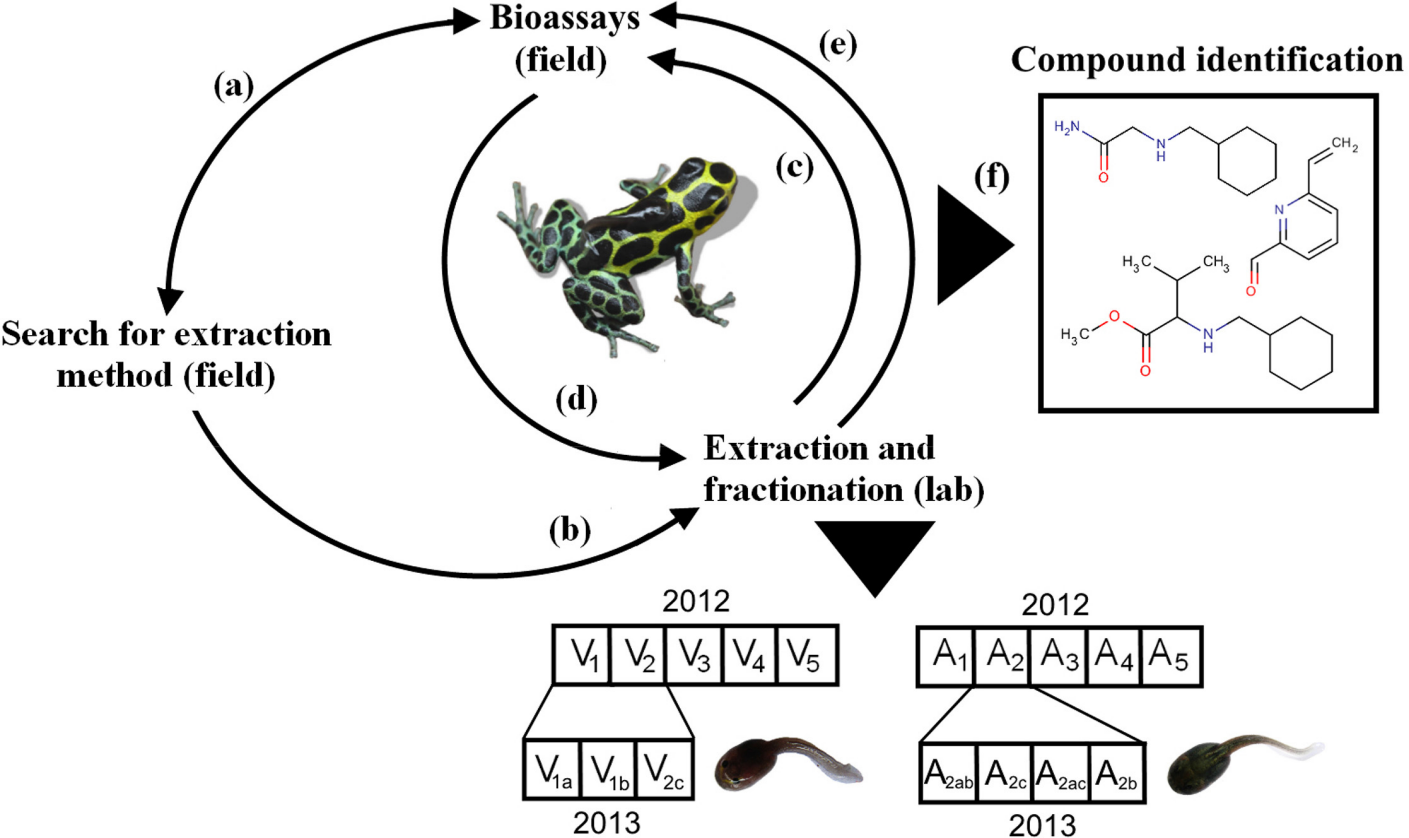
Overview of methods. Steps are numbered in accordance with the text (a—f). V and A stand for fractions of *R*. *variabilis* and *H*. *azureiventris*, respectively. Structural formulas shown in (f) are only examples.

An overview of the succession of the methodical steps is given in Fig 1. Chemicals and analytical instruments used in this study are given in the appendix.

### Ethics statement

The research in Peru was done with permits and according to the guidelines of the Peruvian Ministry of Agriculture (DGFFS) in Lima (authorization No. 0200-2011-AG-DGFFS-DGFFS, 0176-2012-AG-DGFFS-DGFFS and 416-2013-AG-DGFFS-DGFFS; all permits allowed tissue collections of the species, but no tissues were sampled for the project). No animals were killed and all tadpoles were released after the experiment was finished. Study sites were situated at the western side of the Cainarachi River, Región San Martín, Peru: 6°25’53.4”S, 76°17’10.1”W, 6°25’50.3”S, 76°17’30.5”W, 6°25’44.8”S, 76°17’35.5”W.

Captive breeding groups in Germany were legally registered at the nature conservation authority of Trier (Germany). Since we only performed analyses of the water in which tadpoles were kept this was not classified as an animal experiment. Therefore, according to the German animal welfare law and the university representative, Prof. Dr. Thomas Schmitt, formal approval was not necessary.

### (a) Search for a procedure to extract the chemical cues

To find a suitable solid-phase extraction (SPE) sorbent for concentrating the cues from water samples, preliminary experiments were conducted. The goal was to eliminate the active compounds from the tadpole water by sorption to the SPE material and later test these “cleaned” water samples for their activity in bioassays. Successful sorption implies that the active compounds are now in the SPE material and no longer in the water previously used by tadpoles. Accordingly, in following bioassays parental *R*. *variabilis* should not avoid this “cleaned” water for tadpole and egg clutch deposition. The tests of different SPE materials were conducted in-situ in a premontane late-stage secondary forest in the study site mentioned above.

#### (i) Activated carbon

Between 23 March and 8 May 2011 27 tadpoles of each *Ranitomeya variabilis* and *Hyloxalus azureiventris* were collected from their natural habitat and raised in captivity in a wooden hut in the forest serving as a field laboratory during the trials. Each specimen was housed individually in 50 ml of rainwater in identical polypropylene cups. Every other day, we combined the water of the tadpoles (separated by species), refilled the cups with fresh rain water and fed the larvae ad libitum with flaked fish food (Tetra). After finishing the trials or when tadpoles reached Gosner developmental stage 41 [21], they were released back into the wild. The water obtained from the tadpoles was thereafter filtered through approximately 30 g of granular activated carbon (Merck Millipore, 1.5 mm diameter) in order to sorb the odor-producing substances [22].

#### (ii) Chromabond and DSC-18

Two times twenty-five *Ranitomeya variabilis* tadpoles were collected and kept in the field laboratory from 25 March to 8 May 2011 together in one of two containers in 1250 ml of rainwater. Because tadpoles of this species are cannibalistic, each individual was enclosed in a small mesh bag. The mesh was large enough to allow feces to pass, but small enough to prevent physical contact between the tadpoles. A wide opening towards the water surface facilitated the feeding procedure and enabled the animals to take up oxygen. At the bottom of each container a passive sampler was placed (Sartorius membrane filter, 7 cm diameter, 1.2 μm pore size) containing either 1.7 g of Chromabond HR-X (Macherey-Nagel) or 4 g of Discovery DSC-18 (Supelco) to sorb the compounds. Differences in filter content weights were inevitable due to particle size of the sorbents. The water of the trials was collected and replaced every second day followed by feeding of the tadpoles (see (i)). The passive samplers were changed with every third water change. Trials were not conducted with *Hyloxalus azureiventris* because we could not find a sufficient number of tadpoles.

#### (iii) Bioassays in the forest

We established three study sites on the western side of the Cainarachi River at an elevation between 540 and 580 m above sea level. In order to avoid pseudo-replication, i.e. repeated measures on the same specimens, we maintained a minimum distance of 30 m between the sites, which exceeds the known home range size of *Ranitomeya variabilis* [23]. We established artificial phytotelmata at three forest sites. Polypropylene plastic cups (200 ml volume, 10 cm height, 7 cm in diameter), wrapped in dark plastic membranes and with two-thirds of the opening covered, were fixed in pairs to trees at 0.5–1.5 m above ground. Each cup was filled with 25 ml water, one cup per pair with clean rainwater and the other cup with activated carbon, HR-X or C18 treated tadpole water as explained in (i) and (ii). For each treatment we spread 45 cup pairs throughout the three forest sites. They were checked every second day for newly deposited tadpoles and egg clutches of *R*. *variabilis* and both clean and treated water were changed if no depositions were found. Pairs that received a deposition were not used again but another pair was hung up at another tree instead. In order to minimize pseudo-replication, we kept a minimum of 4 m between cup pairs belonging to the same trial and scored only one randomly chosen deposition for any same-day deposition in neighboring cup pairs [18,24].

### (b) Directed chemical extraction and primary fractionation of the chemical compounds

Based on the results of (a) we used Discovery DSC-18 (Supelco) for the extraction and collection of the active chemical compounds from the tadpole water. Although we had no data related to the extraction of chemical compounds produced by *Hyloxalus azureiventris*, we applied our methods in the laboratory to both *Ranitomeya variabilis* and *H*. *azureiventris*.

We used tadpoles from captive breeding colonies at Trier University and kept them from 12 November 2011 to 24 January 2012 at the Department of Effect-Directed Analysis, UFZ Leipzig, where fractionation and chemical analysis were conducted. During this time we had between six and nine *R*. *variabilis* tadpoles and 11 to 23 *H*. *azureiventris* tadpoles. They were kept individually and their water was changed twice a day: six hours (+/-1) a day they were kept in approximately 50 ml of tap water with abundant food supply (see (a)) and 18 hours (+/-1) a day in 10 ml without food. The water without food, containing excretions produced by the tadpoles only, was collected over two days, separated by species, and vacuum filtered for further analysis (Whatman GF/F glass microfiber filters, pore size 0.7 μm). Thereupon a solid phase extraction was conducted, using Discovery DSC-18 (Supelco) as sorbent. The sorbent was preconditioned using dichloromethane (DCM), methanol (MeOH) and bidistilled water before the water sample was passed through the cartridge at a flow rate of about 10.0 ml/min. Once the entire sample had passed through, cartridges were dried under nitrogen and eluted two times with 6 ml of MeOH and DCM each. Elutriates were collected and reduced in volume to dryness in a rotary evaporator. The dried compounds were collected at 4°C and while 214 *R*. *variabilis* and 652 *H*. *azureiventris* samples were kept in the dry form as total samples (V_total_ and A_total_) for later use in the bioassays, 238 *R*. *variabilis* and 406 *H*. *azureiventris* samples were reconstituted in MeOH (10 μL per tadpole sample) for fractionation.

As a control sample to check for background contamination that derived from the fish food, a water sample containing only fish food but no tadpoles was processed by SPE as described above. The LC fractionation was carried out using a Lichrospher 100 RP-18 column (Merck, 250 x 4 mm, 5 μm particle size). A gradient elution was carried out using bidistilled water:methanol 95:5 (eluent A) and water:methanol 5:95 (eluent B) at a flow rate of 1.0 ml/min. The gradient program was 0–4 min 100% A, 4–22 min 0–100% B, 22–25.5 min 100% B, and re-equilibration to 100% A for 5 min. Fractions of samples A and V were collected every minute. Based on LC-HRMS analyses of the sample the one-minute fractions were combined as follows: Fraction A_1-12_/V_1-12_ 10–18 min, A_2-12_/V_2-12_ 18–21 min, A_3-12_/V_3-12_ 21–25 min, A_4-12_/V_4-12_ 25–30 min, A_5-12_/V_5-12_ 30–40 min.

### (c) Bioassays to test which fraction(s) contain the avoided chemical compound(s)

Between 10 February and 18 April 2012 we conducted bioassays in the field to find out which fraction(s) contained the compounds(s) avoided by the frogs. We set up 20 pairs of artificial phytotelmata for each trial as described in (a) and conducted water changes every other day with either clean rainwater or with water containing either the total samples (V_total_ or A_total_) or one of the fractions (V_1-12_ - V_5-12_ or A_1-12_ - A_5-12_) produced in the lab. To mix the dried samples into water, we acted in accordance with the concentrations used in trials with fresh tadpole water before (compare (a) and [18]). We first dissolved our dried samples in 15 μl rainwater per tadpole sample and deep-froze them in measured portions. Those portions were then mixed with rainwater in the field, mixing 15 μl each (i.e. one tadpole that used water in the lab for one day) in 25 ml water. Due to limited chemical compounds, we had to use a smaller amount of water in our cups than before, but the concentration stayed the same. In trials with samples from *R*. *variabilis* we filled 10 ml in each cup and in trials with samples from *H*. *azureiventris* we used 15 ml per cup. Despite this difference, cups were still frequently used for both tadpole and egg clutch depositions and there was no disparity between cups of the same experiments which might have influenced the results [24].

### (d) Second fractionation of the chemical compounds from the tadpole water

During the bioassays after the first fractionation we found several of the tested fractions to be avoided by *Ranitomeya variabilis*. Because each of them was composed of various compounds, a finer fractionation was conducted subsequently. From 18 May to 19 June 2012 and 23 August 2012 to 4 January 2013 water samples of tadpoles from Trier University were collected daily as described in (b). The number of tadpoles varied between five and 23 in *Ranitomeya variabilis* and two and 15 in *Hyloxalus azureiventris*. Chemical compounds were extracted at the Soil Science Department, Trier University, in the way described in (b). Of *R*. *variabilis* 1089 tadpole samples were thereafter not further processed and kept in dried form for a second test of the total sample (V_total_) in the field. The remaining samples (1197 of *R*. *variabilis* and 978 of *H*. *azureiventris*) were further fractionated at UFZ Leipzig. We thereby focused on the fractions that were avoided in the previous bioassays (see results) or where we found similarities between fractions of the two species.

Fractionation of the *R*. *variabilis* samples was conducted in the same manner as described in (b), but the fractions collected every minute were pooled differently and those with no effects in 2012 were not used. Fractions were combined and named as follows: Fraction V_1a-13_ 13–16 min, V_1b-13_ 16–20 min, V_2c-13_ 20–23 min. The numbers in the new names refer to the according fractions of the previous year, while the letters give them a new counting system. Unlike the *R*. *variabilis* samples, the *H*. *azureiventris* samples were at first fractionated and pooled together as in 2012. For a finer fractionation with different selectivity we fractionated fraction A_2-12_ (avoided in 2012, see results) using a Polaris 5 Amide C18 column (Varian, 150 x 4.6 mm, 5 μm particle size). A gradient elution was carried out using bidistilled water:methanol 50:50 (eluent A) and water:methanol 5:95 (eluent B) at a flow rate of 1.0 ml/min. The gradient program was 0–10 min 100% A, 10–15 min 0–100% B, 15–16 min 100% B, and re-equilibration to 100% A for 3.5 min. Fractions of A_2-12_ were collected every minute and combined as follows: Fraction A_2a-13_ 0–7 min, A_2b-13_ 7–15 min, A_2c-13_ 15–20 min.

### (e) Bioassays to test which fraction(s) contain the avoided chemical compound(s)

Bioassays to test which fractions/compounds were avoided by the frogs were conducted in the field, in the same Peruvian sites as mentioned above, with artificial phytotelmata again (compare (a) and (c)). Between 5 February 2013 and 24 May 2013 we offered the fractions and mixtures of fractions to the frogs. The mixtures of the fractions of *H*. *azureiventris* were tested for the case that the compounds were not active in isolated form. We tested the mix of A_2a-13_ and A_2b-13_ (hereafter called A_2ab-13_), A_2c-13_, the mix of A_2a-13_ and A_2c-13_ (hereafter called A_2ac-13_), and A_2b-13_. Thus, with the combination of single and mixed fractions, we were able to determine if a single cue was avoided by the frogs as well. Dried samples were diluted in the same manner as described in (c) with one difference: due to the non- or only nearly significant results for *R*. *variabilis* in 2012 we doubled the concentration of both the total sample as well as the fractions of this species used in 2013. The amount of water used in the cups in the forest was 10 ml each.

### (f) Instrumental analysis and LC-HRMS data analysis

To determine the chemical cues by LC-HRMS, we used the Lichrospher 100 RP-18 column (250 x 4 mm, 5 μm particle size) and gradient described above. The LC was connected to an ESI source and an LTQ Orbitrap XL mass spectrometer. Samples were analyzed in positive and negative full scan mode at a nominal resolving power of 100,000 with data-dependent acquisition of MS/MS spectra at a resolving power of 15,000 triggered by the most and second-most intense peaks in the chromatograms.

The compound identification procedure followed the nontarget screening approach described by Hug et al. [25]. Briefly, fractions were finally analyzed by LC-HRMS using a Kinetex Core-Shell C18 column (100 mm × 3.0 mm; 2.6 μm; Phenomenex) and a gradient elution with water (A) and methanol (B) both containing 0.1% formic acid at a flow rate of 0.2 ml/min. Deconvolution of the full scan chromatograms and peak detection was carried out using the software MZmine 2.9. From the obtained peak lists, those peaks occurring in blank and fish food control samples were removed. For the peaks remaining in active fractions, molecular formulas were calculated based on accurate masses and isotope patterns, and the Chemspider and KEGG databases were searched for all candidate structures of the particular molecular formula. To exclude unlikely candidate structures, we employed MS2 fragmentation prediction and retention time prediction as detailed in Hug et al. [25].

### (g) Statistical analysis

For each treatment tested in (a), (c) and (e) we pooled egg clutch and tadpole depositions because chemical cues of both species are avoided for both deposition types [19]. We compared the deposition frequencies in each water type (clean or treated) using a G-test [26], which is suggested to be better for limited observations [27,28]. Since each dataset had less than 200 observations, we adjusted the G-test following Williams [29]. As a null hypothesis, we assumed that the frequency of deposition events was random (0.5 in either pool).

Because the switch from rainy to dry season is known to have an effect on the deposition decisions of parental *Ranitomeya variabilis* [30], we conducted a changepoint analysis in R (R Development Core Team [31]) with the rainfall measurements taken during each field season, using the package `changepoint´ [32,33]. While there was a significant seasonal change in 2011 (compare [30]), no change could be measured in 2012 and 2013 (bootstrap p > 0.05). All data were taken during the rainy season. We therefore did not have to split our data before analysis.

## Results

### (a) Sorbent to extract the chemical cues

**Fig 2:**
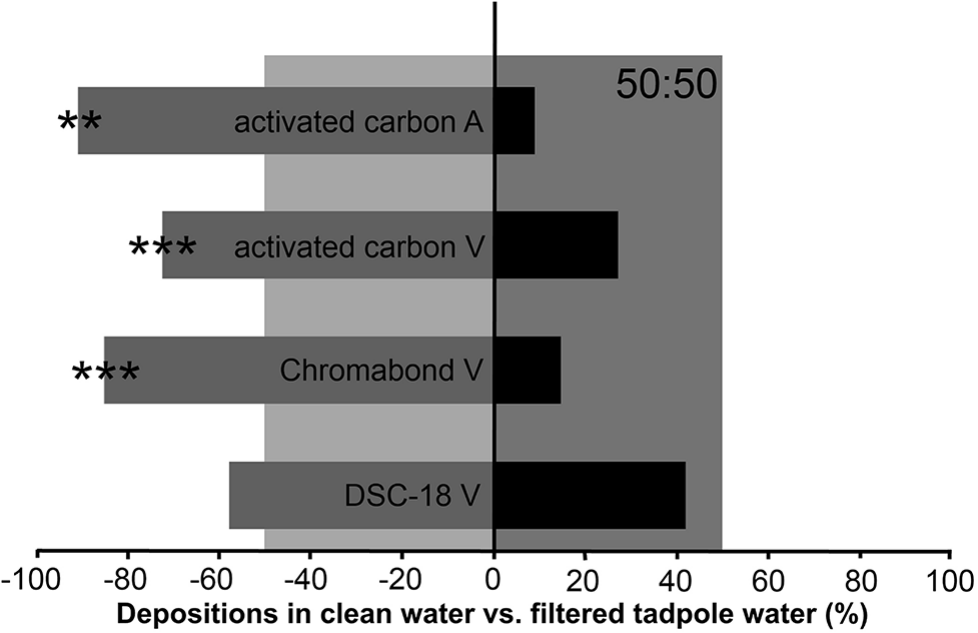
Results of the preliminary trials to find a sorbent to extract the active tadpole compounds. Ratio (in percent) of offspring depositions in clean water (grey narrow bars) and water used by tadpoles and treated with sorbents afterwards (black narrow bars). The expected distribution (50:50) is shown in lighter shades of grey in the background. V and A refer to *R*. *variabilis* and *H*. *azureiventris*, respectively. When frogs showed a significant preference for the clean water we assumed that the treated water still contained tadpole compounds, i.e. the sorbents did not filter them sufficiently out of the water to end the avoidance behavior. Only after the treatment with DSC-18 did frogs not show avoidance of tadpole-treated water. * *p* < 0.05, ** *p* < 0.01, *** *p* < 0.001.

Three different sorbents were tested to extract potential cues or signals from water used by tadpoles of the frogs *Ranitomeya variabilis* and *Hyloxalus azureiventris*. The extraction efficiency was characterized by in-situ avoidance testing. The water used by *R*. *variabilis* or *H*. *azureiventris* was still avoided by parental *R*. *variabilis* for tadpole and egg clutch deposition after extraction with activated carbon (24 in clean and 9 in extracted *R*. *variabilis* water, G = 6.97, p < 0.01; 31 in clean and 3 in filtered *H*. *azureiventris* water, G = 26.45, p < 0.001; Fig 2). Applying passive sampling with Chromabond HR-X could not sufficiently bind the avoided chemical cues either. Parental *R*. *variabilis* still preferred clean over treated water significantly (24 in clean and 4 in passively filtered *R*. *variabilis* water, G = 14.51, p < 0.001). However, water used by *R*. *variabilis* tadpoles extracted with a Discovery DSC-18 passive sampler was not avoided anymore (18 in clean and 13 in passively filtered *R*. *variabilis*-water, G = 0.08, p = 0.37; Fig 2). An influence of the beginning dry season on this result as shown by Schulte and Lötters [30] can be ruled out here. Experiments were stopped a few weeks after the seasonal change (compare changepoint in [30]) and treatments that involved Discovery DSC-18 were not only distributed uniformly after, but also before the changepoint (before changepoint: 4 depositions in clean and 4 in treated water, after changepoint: 14 in clean and 9 in treated water).

### (b) *Ranitomeya variabilis* fractions

**Fig 3:**
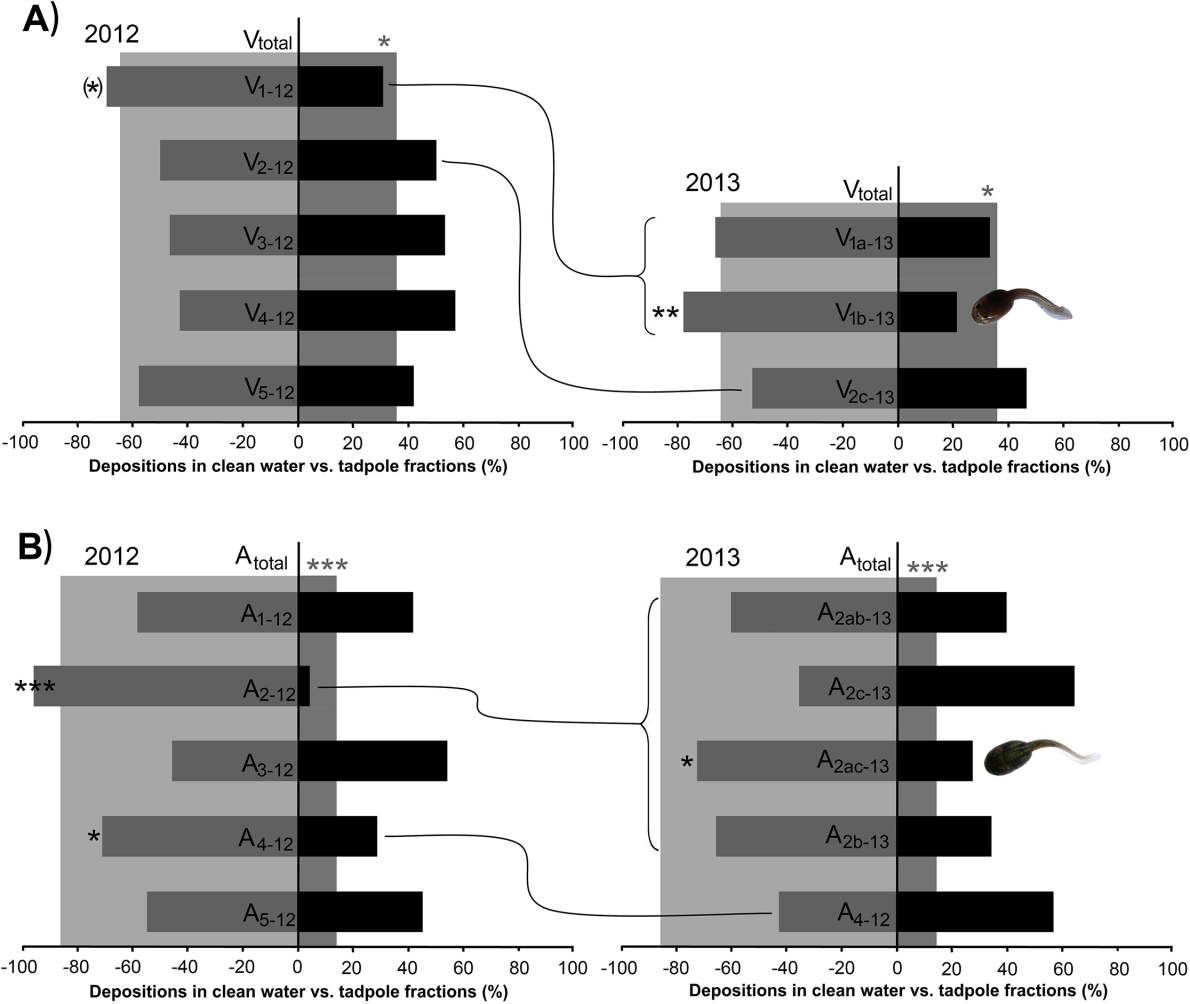
Bioassay results after offering the fractionated compounds of the tadpoles to the frogs. The ratio of offspring depositions in clean water (grey narrow bars) and in water treated with chemically processed (A) *R*. *variabilis* and (B) *H*. *azureiventris* substances (black narrow bars) is shown in percent. V and A stand for *R*. *variabilis* and *H*. *azureiventris*, respectively. The distribution of the total samples (V_total_ and A_total_) is shown with lighter grey shades in the background. Connecting lines leading from results from 2012 to 2013 show which fractions were further processed in 2013 and contain identical compounds. * *p* < 0.05, ** *p* < 0.01, *** *p* < 0.001.

The separation of DSC-18 extracts on C18 provided several fractions which were tested for avoidance in-situ. The results of these bioassays conducted in 2012 and 2013, testing the frogs’ reactions towards the different chemical fractions obtained from *Ranitomeya variabilis* tadpole-water, are shown in Table 1 and Fig 3. None of the fractions tested in 2012 were avoided significantly, but frogs showed a strong tendency towards the clean water over fraction 1 (V_1-12_). When splitting V_1-12_ into smaller fractions in 2013, we found a significant avoidance of fraction b (V_1b-13_). One of the compounds found in V_1b-13_ (see Table 2) was detected in the water of *Hyloxalus azureiventris* as well.

**Table 1:** Pooled egg and tadpole depositions placed by parental *Ranitomeya variabilis* in cups with clean or treated water.

	depositions in experiments with *R*. *variabilis*-fractions					depositions in experiments with *H*.*azureiventris*-fractions				
		clean water	treated water	G-Test	p		clean water	treated water	G-Test	p
2012 + 2013	V_total_	29	16	3.77	0.05	A_total_ *	18	3	11.61	< 0.001
2012	V_1-12_	16	7	3.54	0.06	A_1-12_	11	8	0.46	0.50
	V_2-12_	11	11	0.00	1.00	A_2-12_	21	1	21.87	< 0.001
	V_3-12_	7	8	0.06	0.80	A_3-12_	10	12	0.18	0.67
	V_4-12_	9	12	0.42	0.52	A_4-12_	17	7	4.21	0.04
	V_5-12_	11	8	0.46	0.50	A_5-12_	12	10	0.18	0.67
2013	V_1a-13_	17	15	0.12	0.73	A_2ab-13_	18	12	1.19	0.28
	V_1b-13_	22	6	8.46	< 0.01	A_2c-13_	11	20	2.61	0.11
	V_2c-13_	20	10	3.34	0.07	A_2ac-13_	21	8	5.94	0.01
	-	-	-	-	-	A_2b-13_	21	11	3.13	0.08
	-	-	-	-	-	A_4-13_ **	12	16	0.56	0.45

Treated water contains different fractions (or a total mix) of chemical substances from *R*. *variabilis* (V) or *H*. *azureiventris* (A), tested in 2012 (marked with numbers) and 2013 (marked with the according numbers of 2012 and additional letters), respectively. Deposition frequencies are compared by a G-test.

* A_total_ was only tested in 2012

** A_4-13_ is the only fraction that in 2013 is the same fraction as in 2012 (and it is therefore assigned with a number instead of a letter), but it is missing some cues that could not be found again in 2013.

**Table 2:** Compounds found in fractions avoided in the bioassays by *Ranitomeya variabilis*.

ionisation	*m/z*	retention time	fractions 2012	fractions 2013	retention time of standard method	molecular formula	compound naming
*Ranitomeya variabilis* compounds							
positive	**134.0594**	**14.0**	**V**_**1-12**_	**V**_1**b-13**_	**17.6**	**C**_**8**_ **H**_**7**_ **NO**	**Vari-1**
	**171.1488**	**16.3**			**20.2**	**C**_**9**_ **H**_**18**_ **N**_**2**_ **O**	Vari-2*
negative	151.0400	15.5			nd	nd	
	297.1522	16.2			nd	nd	
	311.1679	16.9			nd	nd	
*Hyloxalus azureiventris* compounds							
positive	**228.1962**	**nd**		**A**_2**c-13**_	**24.1**	**C**_**13**_ **H**_**25**_ **NO**_**2**_	**Azuri-1**
positive	**171.1487**	**16.3**	**A**_**2-12**_	**A**_2**a-13**_	**20.2**	**C**_**9**_ **H**_**18**_ **N**_**2**_ **O**	Azuri-2*
	478.2970	17.7			nd	nd	
negative		16.1			nd	nd	
	527.3033	16.3			nd	nd	

V and A stand for *R*. *variabilis* and *H*. *azureiventris* respectively. Compounds marked with a * were found in fractions of both species; nd = no detection.

### (c) *Hyloxalus azureiventris* fractions

In 2012 parental *Ranitomeya variabilis* avoided both the total sample of the chemically processed *Hyloxalus azureiventris* tadpole-water (A_total_) as well as fractions 2 (A_2-12_) and 4 (A_4-12_) (Table 2, Fig 3). However, the avoidance of fraction 4 was much weaker compared to fraction 2 (compared with a Fisher’s exact test [34]: p = 0.049), as well as compared to the usually very strong general avoidance of *Hyloxalus azureiventris* tadpoles (see [19]; Fisher’s exact test: p = 0.009). Fraction 4 was therefore tested again without further fractionation in the following year and its avoidance could not be confirmed. Fraction 2 however was avoided very strongly in 2012 and was therefore further fractionated in 2013 (Table 2, Fig 3). The mix of the fractions A_2a-13_ and A_2c-13_ (= A_2ac-13_) was avoided significantly. Other fractions or mixes of fractions did not show significant results. One of the compounds found in A_2ac-13_ (compound Azuri-2, see below) was also detected in the water of *R*. *variabilis* (compound Vari-2). Nonetheless, this compound was not active independently (but in combination with Azuri-1 or Vari-1, respectively), because it could also be found in A_2ab-13_ (compare Table 1), which was not avoided by the frogs.

### (d) Identification of chemical cues

In the final active fractions of 2013 a small number of compounds could be detected which were present in the active fractions of 2012 as well (Table 2). Fraction V_1b-13_ from 2013 had two compounds in common with fraction V_1-12_ from 2012. In LC-HRMS these compounds were detected in positive ion mode as ions with m/z 134.0594 (compound Vari-1) and 171.1488 (compound Vari-2), respectively. The latter was also found in fraction A_2a-13_ of fraction-mix A_2ac-13_ (accordingly compound Vari/Azuri-2). Since this compound did not exhibit activity in fraction mix A_2ab-13_, we further analyzed A_2c-13_ (the second fraction in A_2ac-13_). Compound Azuri-1 found in this fraction showed an ion at m/z 228.1962, but it could not be found in A_2-12_.

Based on the accurate mass and isotope patterns and the underlying assumption that protonation in ESI+ occurred, we determined molecular formulas for these compounds (Table 2) and searched the Chemspider database for corresponding structures. For compound Vari-1 (C_8_H_7_NO) 186 candidate structures were found, for compound Vari/Azuri-2 (C_9_H_18_N_2_O) 1653 and for compound Azuri-1 (C_13_H_25_NO_2_) 582.

Based on the approach by Hug et al. [25], we reduced the number of candidate structures by comparing predicted and measured MS2 spectra using the software MetFrag [35]. Candidate structures with a score < 0.8 were excluded. Subsequently, measured and predicted LC retention (as expressed by the chromatographic hydrophobicity index, CHI) was compared based on the method of Ulrich et al. [36] and all candidates removed which were not within ±6 units of CHI.

This candidate reduction procedure resulted in 11 candidates for compound Vari-1 (S1 Table), of which two were considered unlikely due to an anticipated lack of stability. The remaining candidate chemicals include hydroxylated benzonitriles, substituted 1H-pyrrole and pyridine carbaldehydes, and pyridinones. For compound Vari/Azuri-2 14 candidate structures were left (S2 Table), most of them glycinamides. For compound Azuri-1 the candidate reduction procedure did not provide any meaningful MS/MS spectra at different collision energies. Thus, no reduction of candidate structures based on MS/MS data was possible.

## Discussion

Our comparative chemical analysis of the fractions that triggered avoidance behavior in adult *Ranitomeya variabilis* revealed that one compound (C_9_H_18_N_2_O) was produced by tadpoles of both *R*. *variabilis* and *Hyloxalus azureiventris*. This finding would be concordant with our hypothesis (i) that the avoidance behavior of *R*. *variabilis* of both con- and heterospecific tadpoles is based on a reaction to the same chemical compound. The shared compound is most likely a glycinamide, known to contain salty tastants which are shown to act as strong stimuli of the tongue epithelium and the gustatory nerve of bullfrogs (*Lithobates catesbeianus* [37–39]). This finding is well in agreement with our observations and suggests that glycinamides, being recognized by the adults via taste, play a role in intra- and interspecific communication in poison frogs. It might be a compound that tadpoles of all species have in common and is possibly used by adult *R*. *variabilis* to identify if a phytotelm is generally occupied or not. However, for *H*. *azureiventris* the fraction containing this compound could only be shown to be active (i.e. triggering avoidance in the frogs) in combination with one other fraction, but not in another combination. Hence, we suggest that the shared compound is only biologically significant to the frogs in combination with a second compound that gives the frogs more species-specific information about the tadpole species. This leads us to reject hypothesis (i) and support the alternative hypothesis (ii) stating that the active compounds produced by inter- and intraspecific tadpoles are different, as at least one of the avoided compounds is unique in each species (C_8_H_7_NO in *R*. *variabilis* and C_13_H_25_NO_2_ in *H*. *azureiventris*).

Several of the possible structural formulas for the compound unique to *R*. *variabilis* tadpoles are pyridine alkaloids, but none of them has ever been found in anurans. Furthermore, *R*. *variabilis* tadpoles lack alkaloids (pers. comm. Ralph Saporito). With regard to the definition of intraspecific chemical communication between adult *R*. *variabilis* (the receiver) and their tadpoles (the sender), we specified the chemical compounds as chemical cues, because the avoidance is advantageous to the frogs (i.e. their offspring), but not to the tadpoles [18]. However, this definition might be context dependent. While conspecific tadpoles are strongly avoided during the rainy season, with the change to the dry season they are not avoided, but preferred instead. This can be interpreted as a kind of offspring-provision: older tadpoles are fed with younger ones in order to help them survive the dry season [30]. However, since in our study we only focused on the rainy season, we cannot tell if this feeding behavior is triggered by the same chemical compounds as the avoidance behavior, possibly initiating the evolution into chemical signaling (as suggested by Steiger et al. [8]), or if it is triggered by another compound that might be defined as a chemical signal (i.e. pheromone). A switch of preference between rainy and dry season towards *H*. *azureiventris* tadpoles could not been shown [19].

Regarding the communication between adult *R*. *variabilis* and *H*. *azureiventris* tadpoles, the results that at least one of the actively avoided chemical compounds is species specific brings us a step further towards the definition of the interspecifically operating chemicals. We suggest that these species-specific chemical compounds are either chemical signals or chemical cues which are being co-opted for chemical signaling. The reason for this is the obvious benefit the tadpoles of *H*. *azureiventris* receive when *R*. *variabilis* avoid offspring deposition in the same pools. Not only are the predatory tadpoles of *R*. *variabilis* dangerous to the omnivorous, non-predatory *H*. *azureiventris* tadpoles, but also competition in the relatively small water bodies might be a relevant factor [40]. We do not know how the compounds are excreted (e.g. with feces or through the epidermis) or if they are intentionally released by the *H*. *azureiventris* tadpoles. If they are intentionally released, this would be a case of chemical signaling. If accidently released by-products are avoided, this would suggest they are chemical cues. However, since the avoidance of these cues by the receiver (*R*. *variabilis*) turns out to be beneficial to the sender (*H*. *azureiventris*), this might be an example of selection for signaling, as described by Steiger et al. [7] and Wyatt [8].

Assuming the compounds released by *H*. *azureiventris* and recognized by *R*. *variabilis* are an interspecific signal, their ecological context remains unclear. Due to its advantage for the sender, it can be either classified as an allomone (advantageous only to the sender) or a synomone (advantageous to both the sender and the receiver). The fact that the *H*. *azureiventris* tadpoles do not seem to imitate the compounds of the *R*. *variabilis* tadpoles (at least one compound is different) is evidence against a classification as allomones, which are usually replicates of intraspecific pheromones (see below), and rather for the classification as synomones. This classification is further supported by the fact that *R*. *variabilis* is able to distinguish the heterospecific compounds as such and nevertheless avoid them even more intensely than the compounds of its conspecific tadpoles [19].

However, to confirm this classification, the question to be discussed is whether the avoidance of the signal emitted by larval *H*. *azureiventris* is advantageous to *R*. *variabilis* (i.e. its offspring) or not. Since *Ranitomeya* tadpoles are able to feed on *H*. *azureiventris* tadpoles (observation J. L. Brown) the avoidance of this potential prey does not seem to be beneficial to *R*. *variabilis*. This would suggest that the signals are not synomones, but allomones, i.e. beneficial only to the sender. The use of allomones is primarily known from predator-prey systems, where predators lure prey animals with imitations of prey specific pheromones (e.g. [41,42]). This would be the case (in the opposite direction) if *H*. *azureiventris* imitates the cues of the heterospecific tadpoles in order to be avoided by adult *R*. *variabilis*, that is a chemical-based version of Batesian mimicry (which turned out not to be the case). Another possibility are so-called defensive allomones which are toxins used by prey to repel predators (for review see [43]). However, whether those repellents that indicate the toxicity of a prey animal are indeed defined as allomones [9] or rather as synomones [44] is controversial (both definitions were used in the literature to describe avoidance of signals that indicate inedible prey; as allomones: [43,45,46]; as synomones: [47,48]).

Adult poison frogs are a classic example of signaling toxicity. While they use visual signals (aposematic coloration) to warn predators such as birds and mammals [49–52], there is evidence that predators such as spiders, ants and possibly snakes receive chemical signals from the frogs as an indicator of toxicity [53–55]. Due to the fact that toxins (skin alkaloids) are assimilated through food uptake by adult dendrobatid frogs (for review see [56]), it is thought that toxins do not exist in their tadpoles. However, recent research has shown that *Oophaga pumilio* tadpoles can sequester alkaloids from nutritive eggs provided by their parents [57]. But because *H*. *azureiventris* does not egg-feed its offspring, we do not believe that toxins (i.e. repellent allomones/synomones) are involved in the predator-prey relationship between larval *R*. *variabilis* and *H*. *azureiventris*. Furthermore, even the toxicity of adult *H*. *azureiventris* is controversial [58].

Because *H*. *azureiventris* tadpoles grow bigger than *R*. *variabilis* tadpoles and *R*. *variabilis* tadpoles are very small when deposited (Gosner stage 25 [21]), we assume that the avoidance by parental *R*. *variabilis* is associated with competition. Individual small- or medium-sized *R*. *variabilis* larvae are apparently not capable of preying on larger *H*. *azureiventris* tadpoles (N = 11 per species, paired for seven days in 100 ml water contained in artificial pools between 20 May and 2 June 2006; J. L. Brown, unpublished data). We therefore suggest that the chemical signal released by larval *H*. *azureiventris* and avoided by parental *R*. *variabilis* might be a synomone and that the avoidance is advantageous for both species in terms of preventing competition (and for young *H*. *azureiventris*, preventing predation). However, the exact benefits and costs for both species are still to be determined.

There are many examples for synomones between plants and animals, for example between flowers and pollinators (e.g. [59,60]) or between parasite-hosting plants and natural enemies of those parasites (e.g. [61,62]). Synomones between different animal species are less common (aside from predator-warning toxins, depending on the definition, see above). The best known examples are synomones released by sea anemones that initialize their mutualistic relationship with clown fish [63,64]. Chemical communication based on synomones between two species of vertebrates (aside from predator-warning toxins), lacks further examples, to the best of our knowledge. However, by combining ecological studies with chemical analyses, such a communication system might turn out to be more common in mutual competition situations between other vertebrates than is currently known.

## Supporting Information

S1 File Chemicals and analytical instruments. (DOCX)

S1 Table Chemspider candidate structures for unknown compound Vari-1 (C_8_H_7_NO) remaining after candidate selection procedure. (DOCX)

S2 Table Chemspider candidate structures for unknown compound Vari/Azuri-2 (C_9_H_18_N_2_O) remaining after candidate selection procedure. (DOCX)
