# Supplementary material for: Decoding and Discrimination of Chemical Cues and Signals: Avoidance of Predation and Competition during Parental Care Behavior in Sympatric Poison Frogs
Source: PLoS One. 2015 Jul 1;10(7):e0129929. doi: 10.1371/journal.pone.0129929 (PMC4488855; doi:10.1371/journal.pone.0129929)
Supplement: S1 File — (DOCX) [file pone.0129929.s001.docx]

**S1 - Chemicals and analytical instruments**

Methanol (gradient grade), dichloromethane (HPLC grade) and formic acid (analytical reagent grade, 98 %) were supplied by Merck. For detection of chemical cues, we used a Agilent 1200 Liquid chromatography (LC) system consisting of an Autosampler, a binary pump, a column oven and a diode-array detector. The LC system was coupled to a high resolution-high mass accuracy mass spectrometer (HRMS; LTQ Orbitrap XL, Thermo Scientific) using an electrospray ionization (ESI) interface. This system was also used to define the fraction boundaries in the preparative fractionation procedure based on the occurrence of prominent peaks in the HRMS chromatograms. For fractionation, a semipreparative LC system was used consisting of a Rheodyne manual valve, a Varian ProStar 210 Binary Pump System with 25 mL stainless steel pump heads (Varian), an UVD340U diode-array-detector (Dionex) and a Foxy 2000 fraction collector (Teledyne Isco Inc.) controlled by the Chromeleon 6.7 software (Dionex).
