# Supplementary material for: Decoding and Discrimination of Chemical Cues and Signals: Avoidance of Predation and Competition during Parental Care Behavior in Sympatric Poison Frogs
Source: PLoS One. 2015 Jul 1;10(7):e0129929. doi: 10.1371/journal.pone.0129929 (PMC4488855; doi:10.1371/journal.pone.0129929)
Supplement: S1 Table — (DOCX) [file pone.0129929.s002.docx]

**S1 Table.** Chemspider candidate structures for unknown compound Vari-1

(C_8_H_7_NO) remaining after candidate selection procedure.
