# Supplementary material for: Decoding and Discrimination of Chemical Cues and Signals: Avoidance of Predation and Competition during Parental Care Behavior in Sympatric Poison Frogs
Source: PLoS One. 2015 Jul 1;10(7):e0129929. doi: 10.1371/journal.pone.0129929 (PMC4488855; doi:10.1371/journal.pone.0129929)
Supplement: S2 Table — (DOCX) [file pone.0129929.s003.docx]

**S2 Table.** Chemspider candidate structures for unknown compound

Vari/Azuri-2 (C_9_H_18_N_2_O) remaining after candidate selection procedure.
